# Identification of a 4-lncRNA signature predicting prognosis of patients with non-small cell lung cancer: a multicenter study in China

**DOI:** 10.1186/s12967-020-02485-8

**Published:** 2020-08-20

**Authors:** Rui-Qi Wang, Xiao-Ran Long, Chun-Lei Ge, Mei-Yin Zhang, Long Huang, Ning-Ning Zhou, Yi Hu, Rui-Lei Li, Zhen Li, Dong-Ni Chen, Lan-Jun Zhang, Zhe-Sheng Wen, Shi-Juan Mai, Hui-Yun Wang

**Affiliations:** 1 State Key Laboratory of Oncology in South China, Collaborative Innovation Center for Cancer Medicine, Sun Yat-Sen University Cancer Center, Guangzhou, 510060 China; 2 Department of Gynecology and Obstetrics, Renji Hospital, Medical School of Shanghai Jiaotong University, Shanghai, China; 3 Department of Cancer Biotherapy Center, The Third Affiliated Hospital of Kunming Medical University (Tumor Hospital of Yunnan Province), Kunming, Yunnan China; 4 Department of Oncology, The Second Affiliated Hospital of Nanchang University, Nanchang, China; 5 Department of Medical Oncology, Sun Yat-Sen University Cancer Center, Guangzhou, 510060 China; 6 Department of Thoracic Oncology, Sun Yat-Sen University Cancer Center, Guangzhou, 510060 China; 7 State Key Laboratory of Oncology in South China, Sun Yat-Sen University Cancer Center, 651 Dongfeng East Road, Building 2, Rm 704, Guangzhou, 510060 China

**Keywords:** Non-small cell lung cancer (NSCLC), Long non-coding RNA (lncRNA), Signature, Prognosis, TNM stage

## Abstract

**Background:**

Previous findings have indicated that the tumor, nodes, and metastases (TNM) staging system is not sufficient to accurately predict survival outcomes in patients with non-small lung carcinoma (NSCLC). Thus, this study aims to identify a long non-coding RNA (lncRNA) signature for predicting survival in patients with NSCLC and to provide additional prognostic information to TNM staging system.

**Methods:**

Patients with NSCLC were recruited from a hospital and divided into a discovery cohort (n = 194) and validation cohort (n = 172), and detected using a custom lncRNA microarray. Another 73 NSCLC cases obtained from a different hospital (an independent validation cohort) were examined with qRT-PCR. Differentially expressed lncRNAs were determined with the Significance Analysis of Microarrays program, from which lncRNAs associated with survival were identified using Cox regression in the discovery cohort. These prognostic lncRNAs were employed to construct a prognostic signature with a risk-score method. Then, the utility of the prognostic signature was confirmed using the validation cohort and the independent cohort.

**Results:**

In the discovery cohort, we identified 305 lncRNAs that were differentially expressed between the NSCLC tissues and matched, adjacent normal lung tissues, of which 15 are associated with survival; a 4-lncRNA prognostic signature was identified from the 15 survival lncRNAs, which was significantly correlated with survivals of NSCLC patients. This signature was further validated in the validation cohort and independent validation cohort. Moreover, multivariate Cox analysis demonstrates that the 4-lncRNA signature is an independent survival predictor. Then we established a new risk-score model by combining 4-lncRNA signature and TNM staging stage. The receiver operating characteristics (ROC) curve indicates that the prognostic value of the combined model is significantly higher than that of the TNM stage alone, in all the cohorts.

**Conclusions:**

In this study, we identified a 4-lncRNA signature that may be a powerful prognosis biomarker and can provide additional survival information to the TNM staging system.

## Background

Lung cancer is the most common and lethal malignant disease in the world, and approximately about 85% of lung cancer cases are non-small cell lung cancer (NSCLC) [1]. In clinical practice, delayed diagnosis and the lack of effective prognostic biomarkers are two main reasons for poor survival of patients with NSCLC [2, 3]. The 5-year survival rate for patients with late-stage lung cancer and those with stage-I lung cancer is 15% and 83%, respectively [4]. Currently, the treatment strategy and prognosis of lung cancer are mainly determined according to TNM staging system. However, NSCLC patients with the same TNM stage may have a different prognosis [2, 5, 6]. Therefore, an urgent need exists for new biomarkers that can help improve the accuracy of prognosis prediction, which would enhance the quality of life of patients as well as the survival rate [7, 8].

With the development and advancement of high-throughput technologies, numerous investigators have proposed using single genes or gene sets (signatures) as biomarkers for tumor diagnosis, prognosis, disease classification, and personalized treatment. Genomic abnormalities such as DNA mutations, copy-number variations, DNA methylation, and gene expression have been investigated for their usefulness in identifying prognostic biomarkers in patients with NSCLC. High-throughput technologies like microarray and RNA-sequencing (RNA-seq) have enabled simultaneous analysis of hundreds or thousands of genes and their relationships with clinical features, including the survival of patients with cancer, which has led to the discovery of many novel biomarkers (single genes or signatures) for diagnosis, prognosis, and targeted therapy in patients with NSCLC [9, 10]. However, only a few molecular biomarkers have been evaluated in clinical practice (mainly as therapeutic targets) [11] because most of the biomarkers show low accuracy (low sensitivity and/or specificity) [12] or need to be further confirmed with a larger population in an independent validation study [13]. Therefore, more reliable biomarkers are still needed to improve diagnosis, prognosis and personalized therapy for NSCLC patients.

Long non-coding RNAs (lncRNAs) that are expressed at high levels in the body have exhibited superior potential as novel diagnostic or prognostic biomarkers when compared to protein-coding genes, which raises the possibility of identifying more reliable biomarkers for lung cancer [14, 15]. LncRNAs are a type of non-coding RNA that are longer than 200 nucleotides [16, 17]. Accumulating reports have shown that lncRNAs can participate in numerous biological processes, such as the regulation of epigenetic modification, cell cycle progression, and cell differentiation. Growing evidence shows that numerous lncRNAs are significantly deregulated in various types of cancers and play important roles in tumorigenesis [18–20]. An increasing number of lncRNAs have been shown to be dysregulated and involved in lung cancer tumorigenesis, and to be useful as diagnostic or prognostic biomarkers, or as targets for therapy. For example, the lncRNAs MALAT1 and NEAT1 play important roles in lung cancer cell proliferation, cell cycle progression, and apoptosis, as well as tumor progression and prognosis [21–25]. Inhibitors targeting MALAT1 significantly reduced lung cancer metastasis in a mouse model [21]. The prognostic role of lncRNA signatures in NSCLS has been investigated in many reports by using the data downloaded from the Gene Expression Omnibus (GEO) database or The Cancer Genome Atlas (TCGA) database. However, a lncRNA expression profile for especially identifying prognostic signature in a large cohort of NSCLC patients and multicenter study has not been reported yet. Therefore, the prognostic value and the clinical application potentiality of lncRNA signature in NSCLC patients are necessary to be further systematically explored.

In this study, to our knowledge, we performed the first multicenter retrospective study on the prognosis of total 439 NSCLC patients with a custom lncRNA microarray and qRT-PCR. NSCLC patients from South China were randomly divided into a discovery cohort (194 cases) and a validation cohort (172 cases), and those from Southwest China were used as an independent validation cohort (73 cases). A 4-lncRNA signature was established to predict survival of NSCLC patients in the discovery cohort, and was validated in the validation and independent cohorts.

## Methods

### Patients and clinical information

A total of 439 NSCLC cases were collected for this study, and these patients underwent radical resection of lung cancer in the Sun Yat-Sen University Cancer Center (n = 366) and Yunnan Cancer Hospital (n = 73) between 2003 and 2008. Matched cancer tissues and adjacent normal tissues were obtained from each patient recruited in Sun Yat-Sen University Cancer Center. The inclusion criteria for our study were: (i) NSCLC was confirmed by pathological diagnosis and reviewed by 2 experienced pathologists, (ii) the patients did not receive any form of anti-tumor therapy before surgery, (iii) the patients did not die within 1 month after surgery, and (iv) the patient’s sample was preserved at − 80 °C immediately after surgery. The samples collected from the 366 patients enrolled at Sun Yat-Sen University Cancer Center were divided randomly into a discovery cohort (n = 194) and a validation cohort (n = 172). Seventy-three patients with NSCLC were recruited from Yunnan Cancer Hospital (using the inclusion criteria described above) and assigned to an independent validation cohort. Overall Survival (OS) was defined as the time from the date of surgery to the date of death or last follow-up, and disease-free survival (DFS) was defined as the time from the date of surgery to the date of first recurrence or distant metastasis, death, or the last follow-up. The clinicopathological characteristics of the patients in all three cohorts are shown in Table 1. This study was reviewed and approved by the Ethical Committees of Sun Yat-Sen University Cancer Center and Yunnan Cancer Hospital. Written informed consent was obtained from each patient.

**Table 1:** Clinical characteristics of the patients with NSCLC analyzed in the study

Parameters	Discovery cohort (N = 194)	Validation cohort (N = 172)	Independent cohort (N = 73)
Age (X ± SD)	59.2 ± 10.1	59.8 ± 10.2	57.6 ± 9.1
Gender			
Male	144 (74.2%)	136 (79.1%)	52 (71.2%)
Female	50 (25.8%)	36 (20.9%)	21 (28.8%)
TNM stage			
I	87 (44.8%)	74 (43.0%)	23 (31.5%)
II	32 (16.5%)	34 (19.8%)	20 (27.4%)
III	75 (38.7%)	64 (37.2%)	30 (41.1%)
Histological type			
ADC	95 (49.0%)	89 (51.7%)	47 (64.4%)
SCC	88 (45.3%)	76 (44.2%)	26 (35.6%)
ADC/SCC	11 (5.7%)	7 (4.1%)	0 (0.0%)
Tumor size (cm)			
< 5	108 (55.7%)	97 (56.4%)	31 (42.5%)
≥ 5	86 (44.3%)	75 (43.6%)	42 (57.5%)
Differentiation			
Well/moderate	126 (64.9%)	105 (61.0%)	43 (58.9%)
Poor	68 (35.1%)	67 (39.0%)	30 (41.1%)
Lymphatic metastasis			
No	105 (54.1%)	81 (47.1%)	45 (61.6%)
Yes	89 (45.9%)	91 (52.9%)	28 (38.4%)
Follow-up time (month)			
Median (IQR)	37 (24-62)	36 (23-54%)	22 (14-35)
Smoking history			
No	79 (40.7%)	62 (36.0%)	33 (45.2%)
Yes	115 (59.3%)	110 (64.0%)	40 (44.8%)
Family cancer history			
No	161 (83.0%)	151 (87.8%)	71 (97.3%)
Yes	33 (17.0%)	21 (12.2%)	2 (2.7%)

*SD* standard deviation, *ADC* adenocarcinoma, *SCC* squamous cell carcinoma, *IQR* inter-quartile range

### RNA extraction

RNA was extracted from tumor and normal lung tissues using the TRIzol reagent (Invitrogen, Carlsbad, CA, USA) and homogenized with a Bullet Blender (Vortex-Genie 2), according to the manufacturer’s instructions. Briefly, each tissue (100 mg) was mixed with 1 mL TRIzol reagent and homogenized in a Bullet Blender at a 4 °C for 15 min, after which the mixtures were incubated at 25 °C for 5 min. After adding chloroform, the mixtures were violently shaken for 15 s, incubated at room temperature for 10 min, and then centrifuged for 15 min at 4 °C and 14,000 rotations per min. After each supernatant was transferred to a new tube, an equal volume of isopropyl alcohol was added, and the tube contents were mixed. After holding the tubes at room temperature for 10 min, the supernatants were discarded after centrifugation. Each precipitate was washed with 75% alcohol, and then the ethanol was removed after additional centrifugation. After allowing the residual ethanol to evaporate, double-distilled H_2_O was added to dissolve the RNA. Finally, the concentration and quality of each extracted RNA was measured in an ND-1000 spectrophotometer (NanoDrop Technologies), to meet the requirements of the microarray and qRT-PCR experiments.

### Quantitative RT-PCR

Total RNA (1 µg) was reverse transcribed using the GoScript™ Reverse Transcription System (Promega), which includes oligo(dT) primers and random primers for the reverse transcription step, and qPCR was performed using GoTaq^®^ qPCR (Promega) and SYBR Green on a PRISM 7900HT system (Applied Biosystems). Each sample was analyzed in triplicate wells, and reactions without cDNA were included as negative controls. The thermal cycling conditions were as follows: 94 °C at 5 min (for the hot start step), followed by 40 cycles at 94 °C for 15 s and 60 °C for 30 s. The sequences of the primers used in this study are shown in Additional file 1: Table S1. The PCR data were processed by normalizing the median expression value of a given lncRNA to the expression of GAPDH in the same sample. Relative lncRNA-expression levels were quantified using the 2^−ΔΔCt^ method.

### LncRNA microarray fabrication and hybridization

Human lncRNA transcript sequences selected from public lncRNA databases, including the LNCipedia, LncRNAdb, LncRNADisease, and EST databases, were used to design probes for constructing an lncRNA microarray, and 2412 probes were successfully designed. The lncRNA microarray was fabricated in-house and hybridized as described previously [26, 27]. RNA samples obtained from the 366 cancer samples and 100 normal lung tissues in the discovery and validation cohorts, were examined with the lncRNA microarray. Briefly, each probe was mixed with printing buffer to a final concentration of 40 μmol/L and printed in duplicate on the cleaned glass slides (75 × 25 mm). The total RNA 2.0 μg was labeled with 100 nmol/L of Cy5-dUTP (Enzo Life Sciences, New York, USA) in reverse transcription. Then the mixture of labeled RNA sample and 1× hybridization solution was hybridized onto the microarray for 12–18 h at 45 °C. After hybridization, the slides were washed in 1× SSC/1% SDS for 10 min at 45 °C, followed by sequential washing in 2 cycles of 0.5× SSC/0.1% SDS, 2 cycles of 0.2× SSC and 1 cycle of purified water for 1 min at room temperature, respectively, and then dried in a special small centrifuge and scanned using the InnoScan 700A Scanner (Innopsys Inc, France).

### Microarray data processing

The raw microarray data were first processed by subtracting the background signals and then normalized with the quantile method and a log transformation. The log-transformed data were deposited in the GEO database (National Center for Biotechnology Information website), under GEO Accession number GSE143018 (https://www.ncbi.nlm.nih.gov/geo/query/acc.cgi?acc=GSE143018).

To identify differentially expressed lncRNAs between lung cancer tissues and paired normal lung tissues, the Significance Analysis of Microarrays (SAM) program was employed to identify lncRNAs with a fold-change of > 1.25, a *P*-value of < 0.01, and a false-discovery rate (FDR) of < 0.01 (*t* test). Hierarchical-clustering analysis (for classifying the samples in the discovery cohort) was performed using the average-linkage method and uncentered Pearson’s correlation coefficients in MEV software, version 4.2.

### Statistical analysis

Correlations between the 4-lncRNA prognostic signature and clinical characteristics were assessed by Fisher’s exact test and the χ^2^ test, using SPSS software, version 23.0. The prognostic accuracies of the 4-lncRNA signature, the TNM staging system, and the combined-risk model were compared with receiver operating characteristic (ROC) curves, which were generated using MedCalc software, version 11.4.2. The OS and DFS of patients were assessed using the Kaplan–Meier method, and the corresponding graphs were generated using GraphPad Prism software, version 8.0.

The impacts of the lncRNA-expression level and clinical characteristics on DFS and OS were determined using univariate and multivariate Cox-regression models. By employing the risk-score method reported previously [28, 29], 15 lncRNAs were incorporated into different combinations to construct a signature and tested by survival analysis, and the lncRNAs were gradually subtracted from the combinations to obtain a final 4-lncRNA signature with the greatest prognostic value.

## Results

### Detection of lncRNA-expression profiles in NSCLC tissues from the discovery cohort, using a custom microarray

The 366 patients with NSCLC from Sun Yat-Sen University Cancer Center in Southern China were randomly divided into a discovery cohort and a validation cohort. The clinical characteristics of these patients are shown in Table 1. We first detected the lncRNA-expression profiles in 194 NSCLC samples and 100 matched normal lung tissues in the discovery cohort, using an in-house generated lncRNA microarray containing 2412 human lncRNA probes. After subtracting the background signals, and normalizing and log-transforming the microarray data, we analyzed the lncRNA-expression profiles with the SAM program and Student’s *t* test, and identified 305 differentially expressed lncRNAs between the NSCLC tissues and adjacent normal lung tissues (FDR = 0 and fold-change > 1.25), of which 138 lncRNAs were upregulated and 167 were down-regulated in the NSCLC tissues (Additional file 1: Fig. S1 and Table S2). The log-transformed microarray data were submitted and deposited in the GEO database.

**Fig. 1:**
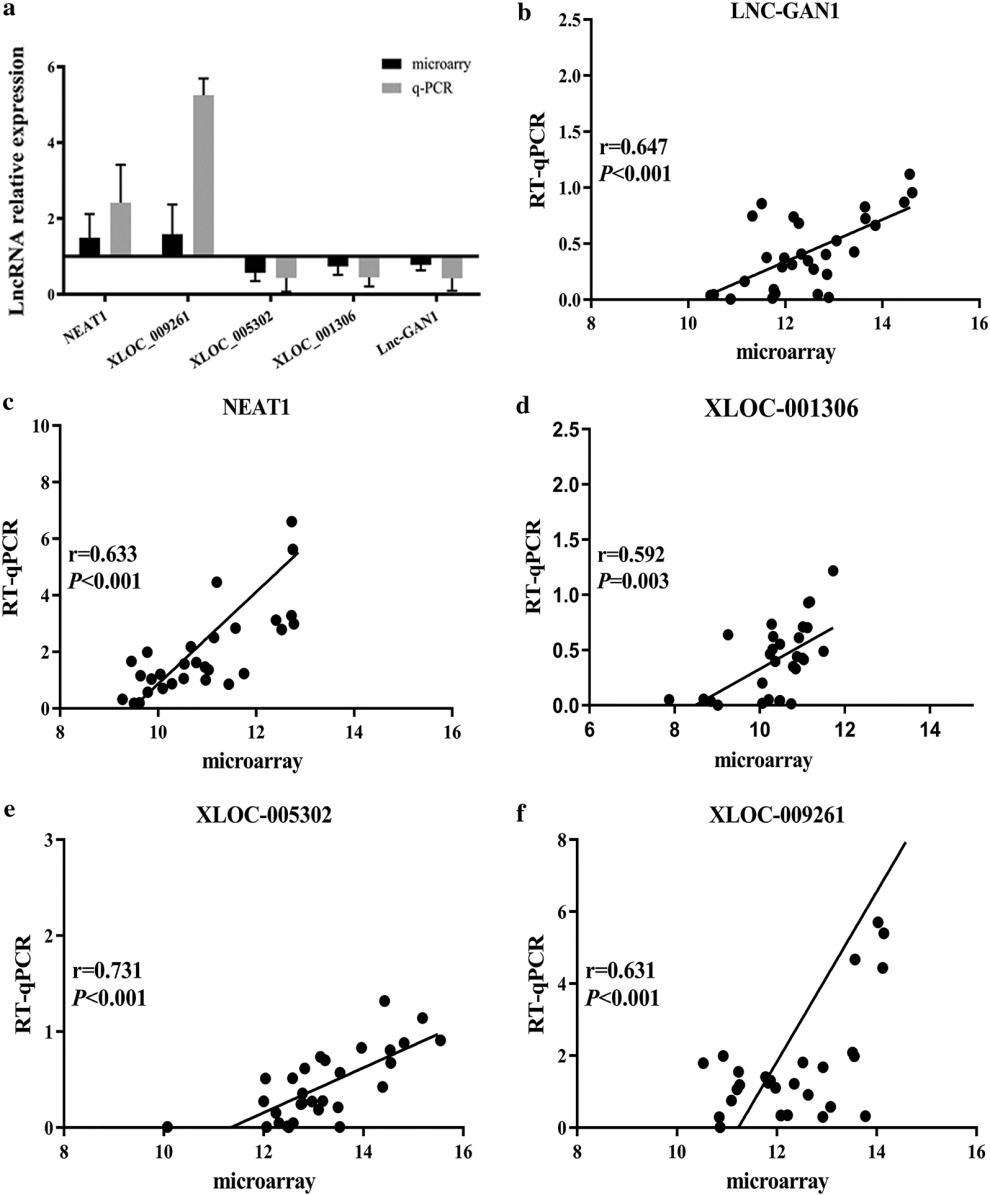
Comparison of microarray data with qRT-PCR data. To confirm the microarray data are reliable and reproducible, five lncRNAs were measured by real-time quantitative RT-PCR in 30 pairs of lung cancer and matched normal lung tissues. **a** The expression levels of 5 lncRNAs detected by microarray were consistent with those measured by qRT-PCR. **b**–**f** Significant correlations were found between the expression levels of 5 lncRNAs detected by real-time qPCR and by the microarray (Pearson correlation, *P* < 0.001)

To confirm the reliability and repeatability of the microarray results, 5 out of 15 prognostic lncRNAs were selected for qRT-PCR analysis with 30 pairs of samples that were randomly selected from the discovery cohort. Of these 5 lncRNAs, 2 (NEAT1 and XLOC_009261) were up-regulated and 3 (XLOC_005302, XLOC_001306, and lnc-GAN1) were down-regulated in the lung cancer tissues, compared with that in the normal lung tissues. The expression-level ratios of the 5 lncRNAs in cancer tissues versus adjacent tissues detected by qRT-PCR were consistent with the microarray results (Fig. 1a) and significant correlations were found between the qRT-PCR and microarray data for the 5 lncRNAs (Fig. 1b–f). These results reveal that the lncRNA-expression levels detected with the lncRNA microarray are reliable and reproducible, which can be used for further analysis.

### Identification of a 4-lncRNA prognostic signature for NSCLC patients in the discovery cohort

To elucidate the prognostic significance of lncRNAs in NSCLC, we conducted univariate Cox regression analysis on all 305 differentially expressed lncRNAs in the discovery cohort. Based on the threshold of *P*-value<0.05, 15 lncRNAs were significantly associated with OS in the NSCLC patients (Table 2), of which 6 lncRNAs were risky and 9 lncRNAs were protective.

**Table 2:** Summary of 15 lncRNAs associated with overall survival of NSCLC patients in the discovery cohort

No	LncRNA	Weight	P value	HR (95% CI)	Putative function
1	BF768381	0.168	0.048	1.183 (1.001–1.390)	High-risk
2	DD3	0.212	0.035	1.236 (1.015–1.500)	High-risk
3	BF944729	0.228	0.045	1.255 (1.005–1.560)	High-risk
4	SRG1	0.439	0.006	1.552 (1.136–2.120)	High-risk
5	**NEAT1**	0.412	0.003	1.510 (1.154–1.970)	High-risk
6	Zeb2NAT	0.344	0.019	1.411 (1.057–1.880)	High-risk
7	ASLNC03555	− 0.574	0.025	0.563 (0.342–0.920)	Protective
8	ASLNC09137	− 0.488	0.025	0.614 (0.401–0.940)	Protective
9	GSO_1539211_377	− 0.578	0.039	0.561 (0.324–0.970)	Protective
10	GSO_1539832_035	− 0.486	0.041	0.615 (0.386–0.980)	Protective
11	**Lnc-GAN1**	− 0.349	0.048	0.705 (0.499–0.990)	Protective
12	GSO_1539211_480	− 0.446	0.007	0.640 (0.463–0.880)	Protective
13	**ASLNC11245**	− 1.269	0.000	0.281 (0.143–0.550)	Protective
14	BF375442	− 0.348	0.026	0.706 (0.520–0.950)	Protective
15	**GSO_1539832_023**	− 0.503	0.010	0.605 (0.412–0.880)	Protective

To determine an optimal lncRNA combination (signature) for predicting the survival outcomes of patients with NSCLC, we employed the 15 lncRNAs associated with survival to establish a prognostic signature with a risk-score method, as previously reported [28, 29]. Using this method, we established a 4-lncRNA signature with the highest prognostic power, consisting of NEAT1, lnc-GAN1, ASLNC11245, and GSO_1539832_023. Based on the expression levels of the 4 lncRNAs (measured by microarray analysis and weighted by their corresponding regression coefficients derived from univariate Cox-regression analysis), the risk scores were calculated as follows:

$$\begin{aligned} {\text{Risk score}} & = \left( {0.412 \times {\text{NEAT1 level}}} \right) + \left( { - 0. 3 4 9\times {\text{lnc - GAN1 level}}} \right) \\ & \quad + \left( { - 1. 2 6 9\times {\text{ASLNC11245 level}}} \right) + \left( { - 0. 50 3\times {\text{GSO}}\_ 1 5 3 9 8 3 2\_0 2 3 {\text{ level}}} \right). \\ \end{aligned}$$

**Fig. 2:**
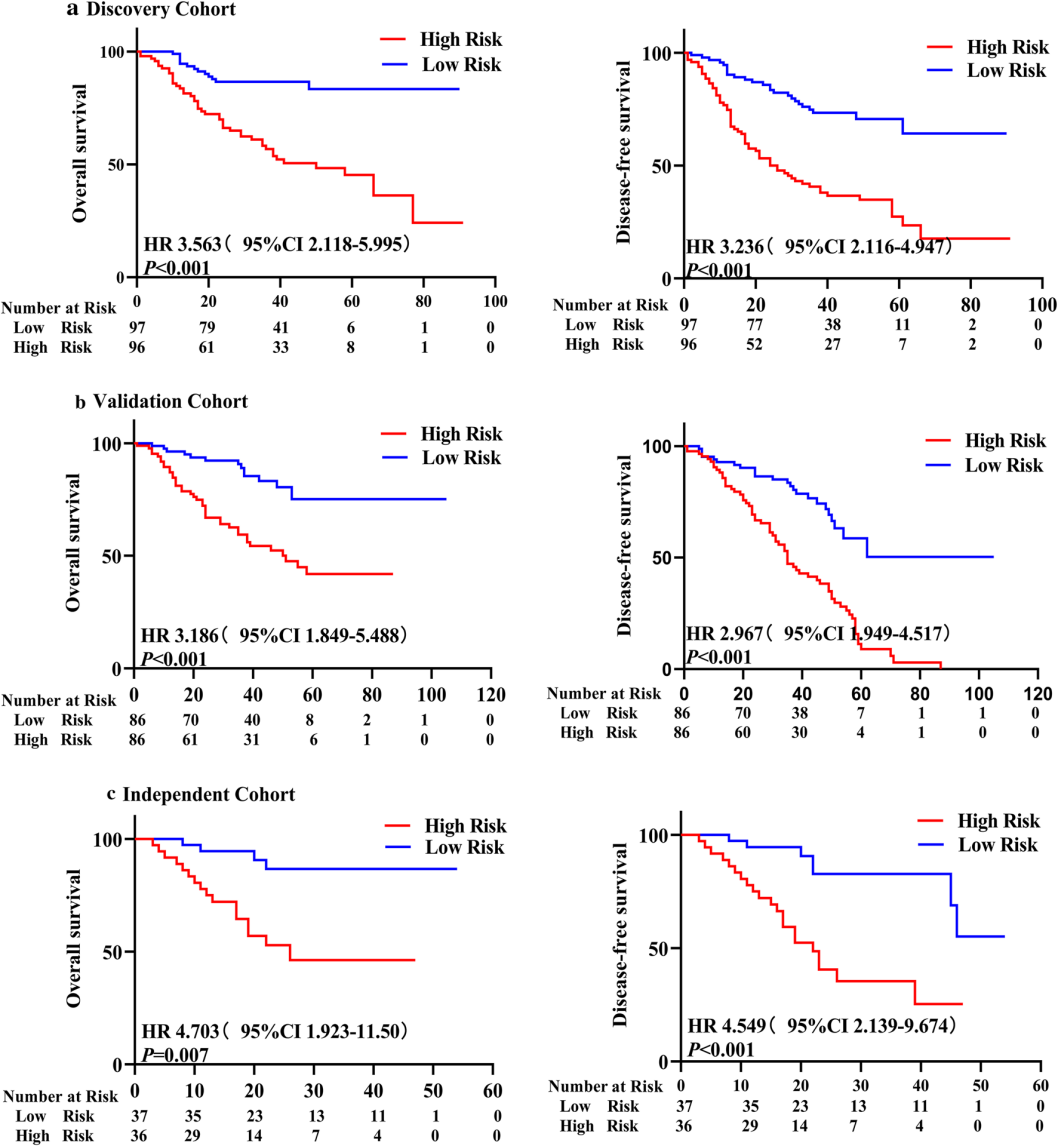
The 4-lncRNA signature as a powerful predictor for OS and DFS of patients with NSCLC in the 3 cohorts. Patients with NSCLC were divided into high- and low-risk groups, based on the 4-lncRNA signature risk, and analyzed with Kaplan–Meier survival curves. Patients with high-risk had significantly worse OS (left panel) and DFS (right panel) in (**a**) the discovery cohort (n = 194), **b** the validation cohort (n = 172) and **c** the independent cohort (n = 73)

The risk-score formula was used to calculate risk scores for each patient, who were divided into high- and low-risk groups according to median risk score. Kaplan–Meier-survival analysis showed that patients in the high-risk group had remarkably lower OS and DFS rates than those in the low-risk group (Fig. 2a), implying that this prognostic signature is potentially highly effective for predicting the survival of patients with NSCLC.

### Validation of the 4-lncRNA prognostic signature in patients with NSCLC from a multicenter registry

To verify the prognostic value of the 4-lncRNA signature identified in the discovery cohort, we attempted to validate it with NSCLC patients from two different geographical locations, where one cohort was used as an internal validation cohort, and the other was used as an independent validation cohort. First, we tested the 4-lncRNA signature with the internal validation cohort (n = 172 NSCLC samples) acquired from the same center as the discovery cohort in southern China. The NSCLC samples in the internal validation cohort were analyzed using the same lncRNA microarray and risk-score formula that was used for the discovery cohort. Based on the risk scores, patients in the internal validation cohort were classified into high-risk and low-risk groups. Survival analysis showed that patients in the high-risk group had significantly lower OS and DFS rates than those in the low-risk group (Fig. 2b), which was consistent with the results obtained in the discovery cohort.

Second, we tested the 4-lncRNA prognostic signature with another 73 NSCLC samples (as an independent validation cohort) obtained from another medical center in southwestern China and detected the expression of the 4 lncRNAs using qRT-PCR. Then, univariate Cox-regression analysis was performed on the 4 lncRNAs, and a risk-score formula was constructed with the same method used in the discovery cohort:

$$\begin{aligned} {\text{Risk score}} & = (0. 2 9 7\times {\text{NEAT1}}\;{\text{level}}) + ( - 0. 2 5 9\times {\text{lnc - GAN1}}\;{\text{level}}) \\ & \quad + ( - 0. 70 6\times {\text{ASLNC11245}}\;{\text{level}}) + ( - 0. 1 5 3\times {\text{GSO}}\_ 1 5 3 9 8 3 2\_0 2 3\;{\text{level}}). \\ \end{aligned}$$

We calculated the risk score for each patient with the new formula (shown immediately above) in the independent validation cohort. By applying the median risk score as the cutoff point, patients were categorized into high- and low-risk groups. As shown in Fig. 2c, the OS and DFS rates of patients with NSCLC in the high-risk group were significantly lower than those in the low-risk group, which was in concordance with the results obtained from the discovery and internal validation cohorts. The above results demonstrated that the 4-lncRNA signature is correlated significantly with the prognosis of patients with NSCLC from a multicenter cohort in different geographical regions, suggesting that the 4-lncRNA signature is a new and powerful prognostic biomarker for patients with NSCLC from different regions of China.

### The 4-lncRNA prognostic signature was independent of the TNM staging system

**Fig. 3:**
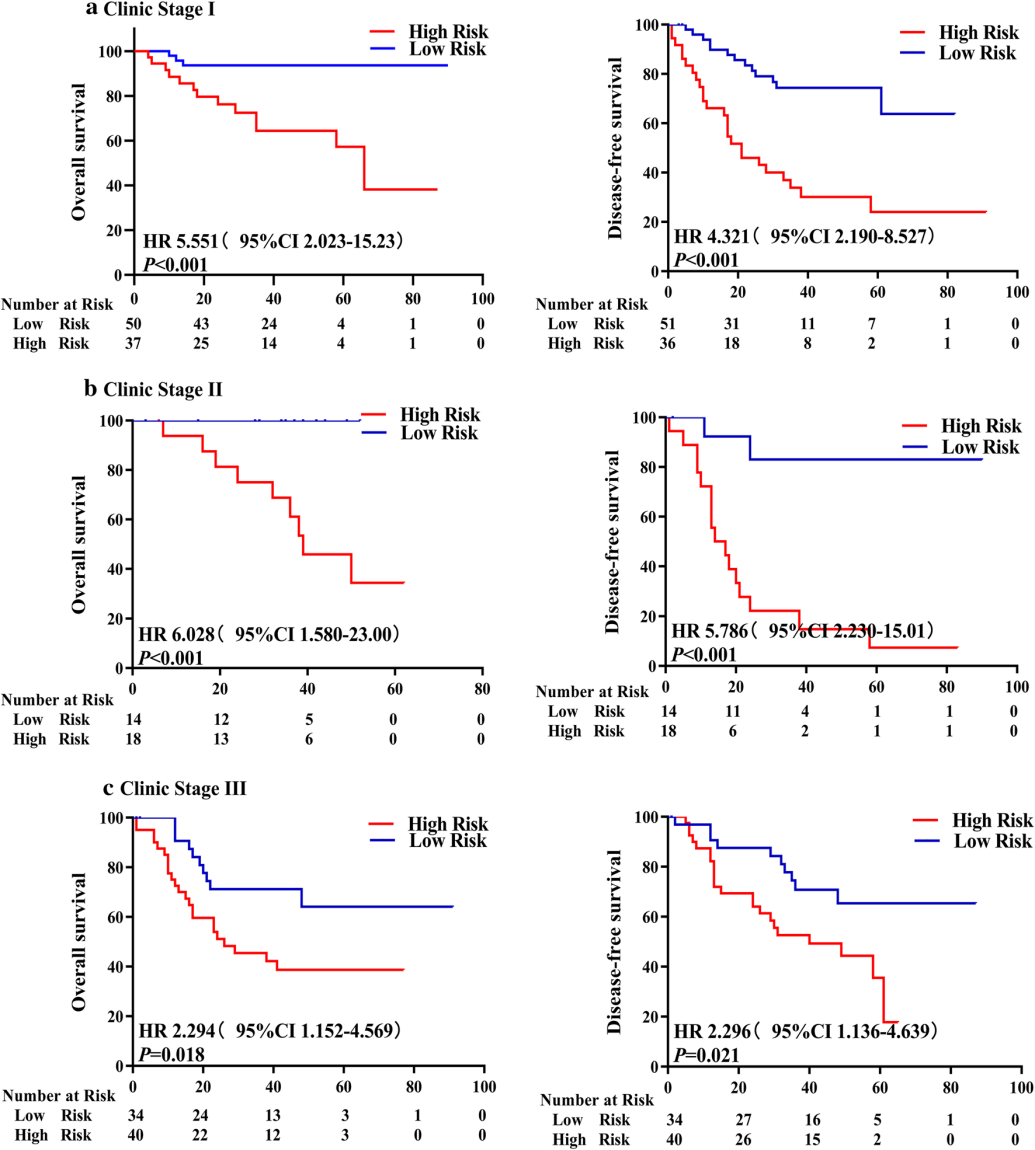
The 4-lncRNA signature predicted different survivals rates in patients with NSCLC at the same TNM stage. Based on the 4-lncRNA signature risk score, patients with NSCLC at the same stage were divided into high- and low-risk groups. Kaplan–Meier survival analysis was performed to estimate patients’ survival rate in the discovery cohort. NSCLC patients with high risk (based on the 4-lncRNA signature) showed significantly poorer OS (left panel) and DFS (right panel) rates than those in low-risk group at **a** stage I (n = 87), **b** stage II (n = 32) and **c** stage III (n = 84)

To gain deeper insight into the clinical significance of the 4-lncRNA signature, we first conducted a correlation analysis between the signature and any associated clinical characteristics. The results showed that the 4-lncRNA signature did not correlate with any clinical characteristics in the 3 cohorts (Table 3), implying that the signature was independent of the clinical characteristics. Then, we carried out a univariate Cox-regression analysis of the signature and clinical characteristics. The results revealed that only the 4-lncRNA signature and TNM stage were associated with the OS (Table 4) and DFS (Table 5) rates of patients with NSCLC in all the 3 cohorts, providing further evidence that the 4-lncRNA signature is a useful prognostic indicator. Finally, we performed a multivariate Cox-regression analysis on the 4-lncRNA signature and all clinical characteristics. After adjustment for other clinicopathological variables, both the 4-lncRNA signature and the TNM stage correlated significantly with OS and DFS rates of patients in all the 3 cohorts, whereas other factors did not (Table 6). To further confirm the utility of the 4-lncRNA signature as an independent predictive factor for survival, we performed a stratified analysis of patients at three different TNM stages with the 4-lncRNA prognostic signature. Patients in the same TNM stage (stage I, II, or III) were divided into high- or low-risk subgroups, based on the risk scores generated with the 4-lncRNA prognostic signature. The results showed that NSCLC patients with high-risk scores generally had significantly lower OS and DFS rates than those with low-risk scores (Fig. 3) in stage I, II, or III, indicating that the prognostic 4-lncRNA signature is performed independently of the TNM staging system. Collectively, these results indicated that the 4-lncRNA signature is a powerful and independent prognostic indicator for patients with NSCLC.

**Table 3:** The relationship between 4-lncRNA signature and Clinical characteristics in the three NSCLC patient cohorts

Characteristics	Discovery cohort (N = 194)			Validation cohort (N = 172)			Independent cohort (N = 73)		
	Low-risk	High-risk	*P* value	Low-risk	High-risk	*P* value	Low-risk	High-risk	*P* value
	n (%)	n (%)		n (%)	n (%)		n (%)	n (%)	
Age									
≥ 60	52 (53.6)	49 (50.5)	0.706	49 (57.0)	46 (53.5)	0.816	19 (51.4)	21 (58.3)	0.493
< 60	45 (46.4)	48 (49.5)		37 (43.0)	40 (46.5)		18 (48.6)	15 (41.7)	
Gender									
Male	82 (84.5)	72 (74.2)	0.269	67 (77.9)	69 (80.2)	0.374	29 (78.4)	23 (63.9)	0.290
Female	15 (15.5)	25 (25.8)		19 (22.1)	17 (19.8)		8 (21.6)	13 (36.1)	
TNM stage									
I	46 (47.4)	41 (42.3)	0.637	39 (45.3)	35 (40.7)	0.702	13 (35.1)	10 (27.8)	0.518
II	11 (11.3)	21 (21.6)		18 (21.0)	16 (18.6)		8 (21.6)	12 (33.3)	
III	40 (41.2)	35 (36.1)		29 (33.7)	35 (40.7)		16 (43.2)	14 (38.9)	
Histological type									
ADC	55 (56.7)	40 (41.2)	0.304	39 (45.3)	50 (58.1)	0.297	25 (67.6)	22 (61.1)	0.451
SCC	39 (40.2)	49 (50.5)		40 (46.5)	36 (41.9)		12 (32.4)	14 (38.9)	
ADC/SCC	3 (3.1)	8 (8.3)		7 (8.2)	0 (0.0)		0 (0.0)	0 (0.0)	
Tumor size (cm)									
< 5	59 (60.8)	49 (50.5)	0.332	46 (53.5)	51 (59.3)	0.573	13 (35.1)	18 (50.0)	0.197
≥ 5	38 (39.2)	48 (49.5)		40 (46.5)	35 (40.7)		24 (64.9)	18 (50.0)	
Differentiation									
Well/moderate	58 (59.8)	68 (70.1)	0.402	44 (51.2)	61 (70.9)	0.203	19 (51.4)	24 (66.7)	0.310
Poor	39 (40.2)	29 (29.9)		42 (48.8)	25 (29.1)		18 (48.6)	12 (33.3)	
Lymph metastasis									
No	47 (48.5)	58 (59.8)	0.257	39 (45.3)	42 (48.8)	0.574	21 (56.8)	24 (66.7)	0.297
Yes	50 (51.5)	39 (40.2)		47(54.7)	44 (51.2)		16 (43.2)	12 (33.3)	
Smoking history									
No	36 (37.1)	43 (44.3)	0.503	29 (33.7)	33 (38.4)	0.692	15 (40.5)	18 (50.0)	0.307
Yes	61 (62.9)	54 (55.7)		57 (66.3)	53 (61.6)		22 (59.5)	18 (50.0)	
Fam. cancer hist.									
No	84 (86.6)	77 (79.3)	0.396	73 (75.3)	78 (90.7)	0.417	36 (97.3)	35 (97.2)	0.664
Yes	13 (13.4)	20 (20.7)		13 (13.4)	8 (9.3)		1 (2.7)	1 (2.8)	

*Fam. cancer hist.* Family cancer history

**Table 4:** Univariate Cox regression analysis of the impact of the lncRNA signature and other clinicopathological features on OS in the three NSCLC patient cohorts

Parameters	Training cohort		Validation cohort		Independent cohort	
	Hazard ratio (95% CI)	*P* value	Hazard ratio (95% CI)	*P* value	Hazard ratio (95% CI)	*P* value
Signature						
(High vs low)	3.20 (0.58–1.65)	*< 0.001*	2.84 (1.59–5.07)	*< 0.001*	2.84 (1.59–5.07)	*0.009*
Age						
(≥ 60 vs < 60)	1.24 (0.73–2.09)	0.417	1.13 (0.67–1.91)	0.330	0.88 (0.36–2.13)	0.782
Gender						
(Male vs female)	0.86 (0.48–1.52)	0.619	1.24 (0.63–2.41)	0.050	0.98 (0.37–2.57)	0.978
TNM stages						
(III vs II vs I)	1.67 (1.28–2.19)	*< 0.001*	1.70 (1.30–2.23)	*0.001*	1.74 (1.04–2.89)	*0.031*
Histological type						
(ADC vs SCC)	1.30 (0.74–2.29)	0.346	1.39 (0.82–2.36)	0.589	0.53 (0.15–1.83)	0.320
Tumor size						
(≥ 5 cm vs < 5 cm)	1.17 (0.69–1.98)	0.545	1.15 (0.68–1.95)	*0.017*	3.00 (1.00–9.00)	*0.048*
Differentiation						
(Poor vs well/mod)	0.98 (0.58–1.65)	0.951	1.43 (0.85–2.42)	0.079	1.80 (0.74–4.32)	0.188
Lymph metastasis						
(Yes vs no)	1.44 (0.86–2.43)	0.163	0.73 (0.43–1.24)	*0.025*	1.38 (0.46–4.14)	0.561
Smoking history						
(Yes vs no)	0.91 (0.54–1.54)	0.736	1.63 (0.92–2.91)	*0.024*	1.11 (0.46–2.68)	0.812
Fam. cancer hist.						
(Yes vs no)	1.04 (0.52–2.06)	0.899	1.18 (0.58–2.42)	0.580	0.47 (2.97–7.64)	0.618

Italic *P* values represent the statistic significance

*Fam. cancer hist.* Family cancer history

**Table 5:** Univariate Cox regression analysis of the impact of lncRNA signature and other clinicopathological features on DFS in the three NSCLC patient cohorts

Parameters	Training group		Validation group		Independent group	
	Hazard ratio (95% CI)	*P* value	Hazard ratio (95% CI)	*P* value	Hazard ratio (95% CI)	*P* value
Signature						
(High vs low)	2.61 (1.50–4.56)	*< 0.001*	3.21 (1.80–5.71)	*< 0.001*	2.18 (1.10–4.34)	*0.025*
Age						
(≥ 60 vs < 60)	1.30 (0.76–2.21)	0.330	1.30 (0.76–2.21)	0.510	1.19 (0.60–2.35)	0.599
Gender						
(Male vs female)	0.57 (0.33–1.00)	0.050	0.97 (0.53–1.78)	0.945	0.77 (0.37–1.60)	0.496
TNM stages						
(III vs II vs I)	1.55 (1.18–2.04)	*0.001*	1.70 (1.29–2.25)	*< 0.001*	1.46 (1.00–2.12)	*0.045*
Histological type						
(ADC vs SCC)	0.83 (0.44–1.59)	0.589	1.48 (0.88–2.49)	0.133	1.02 (0.45–2.29)	0.954
Tumor size						
(≥ 5 cm vs < 5 cm)	1.89 (1.12–3.22)	*0.017*	1.57 (0.94–2.61)	0.082	1.92 (0.92–4.03)	0.081
Differentiation						
(Poor vs well/moderate)	0.62 (0.36–1.05)	0.079	1.22 (0.73–2.04)	0.427	1.29 (0.65–2.57)	0.453
Lymph metastasis						
(Yes vs no)	1.82 (1.07–3.10)	*0.025*	1.72 (1.01–2.91)	*0.042*	2.13 (0.87–5.23)	0.095
Smoking history						
(Yes vs no)	0.54 (0.32–0.92)	*0.024*	1.15 (0.68–1.95)	0.586	1.00 (0.51–1.97)	0.989
Fam. cancer hist.						
(Yes vs no)	0.80 (0.38–1.71)	0.580	0.69 (0.31–1.53)	0.369	1.23 (0.16–9.12)	0.834

Italic *P* values represent the statistic significance

*Fam. cancer hist.* Family cancer history

**Table 6:** Multivariate Cox regression analysis of the impact of lncRNA signature and clinicopathological features on OS and DFS in the three NSCLC patient cohorts

Dataset	Parameters	Overall survival		Disease-free survival	
		Hazard ratio (95% CI)	*P* value	Hazard ratio (95% CI)	*P* value
Training	Signature	3.18 (1.62–6.23)	*0.001*	2.17 (1.35–3.47)	*0.001*
	Age	1.07 (0.78–1.45)	0.401	1.03 (0.76–1.40)	0.845
	Gender	1.61 (0.57–4.51)	0.365	1.42 (0.54–3.73)	0.474
	TNM stages	1.61 (1.09–2.15)	*0.008*	1.47 (1.06–2.05)	*0.022*
	Histological types	0.99 (0.61–1.61)	0.965	0.94 (0.58–1.51)	0.783
	Tumor sizes	0.91 (0.47–1.73)	0.767	0.86 (0.45–1.64)	0.657
	Differentiation	1.18 (0.78–1.78)	0.43	1.12 (0.75–1.68)	0.587
	Pleural invasion	1.60 (0.85–3.01)	0.147	1.79 (0.96–3.35)	0.068
	Vascular invasion	2.17 (0.75–6.28)	0.154	1.86 (0.65–5.34)	0.248
	Smoking history	2.94 (1.15–7.50)	*0.024*	2.60 (1.09–6.24)	*0.032*
	Fam. cancer hist.	0.58 (0.25–1.37)	0.218	0.53 (0.23–1.25)	0.146
Validation	Signature	2.41 (1.47–3.97)	*0.001*	2.49 (1.53–4.05)	*<0.001*
	Age	1.14 (0.87–1.49)	0.359	1.13 (0.87–1.48)	0.349
	Gender	0.64 (0.32–1.27)	0.201	0.79 (0.41–1.55)	0.498
	TNM stages	1.40 (1.03–1.91)	*0.031*	1.40 (1.04–1.88)	*0.026*
	Histological types	0.92 (0.62–1.38)	0.697	0.94 (0.63–1.4)	0.763
	Tumor sizes	1.41 (0.80–2.49)	0.240	1.27 (0.73–2.23)	0.402
	Differentiation	0.89 (0.59–1.32)	0.552	0.88 (0.60–1.31)	0.537
	Pleural invasion	1.42 (0.85–2.39)	0.185	1.51 (0.90–2.52)	0.116
	Vascular invasion	5.40 (1.73–16.8)	*0.004*	4.91 (1.59–15.17)	*0.006*
	Smoking history	0.54 (0.28–1.04)	0.064	0.52 (0.27–1.00)	*0.050*
	Fam. cancer hist.	1.22 (0.66–2.28)	0.521	1.48 (0.81–2.70)	0.206
Independent	Signature	1.88 (1.15–3.08)	*0.012*	1.80 (1.14–2.84)	*0.012*
	Age	1.00 (0.70–1.42)	0.988	1.19 (0.86–1.66)	0.294
	Gender	0.80 (0.46–1.40)	0.430	0.70 (0.42–1.18)	0.183
	TNM stages	1.80 (1.28–2.54)	*0.001*	1.69 (1.24–2.30)	*0.001*
	Histological types	1.26 (0.74–2.13)	0.395	1.12 (0.69–1.84)	0.640
	Tumor sizes	1.78 (1.06–2.97)	*0.028*	1.92 (1.19–3.09)	*0.008*
	Differentiation	1.66 (1.03–2.66)	*0.037*	1.81 (1.16–2.83)	*0.009*
	Pleural invasion	1.26 (0.68–2.33)	0.466	1.59 (0.87–2.91)	0.130
	Vascular invasion	1.75 (0.39–7.92)	0.468	2.81 (0.79–9.98)	0.110

Italic *P* values represent the statistic significance

*Fam. cancer hist.* Family cancer history

### The 4-lncRNA signature provids additional prognostic information to the TNM staging system in patients with NSCLC

**Fig. 4:**
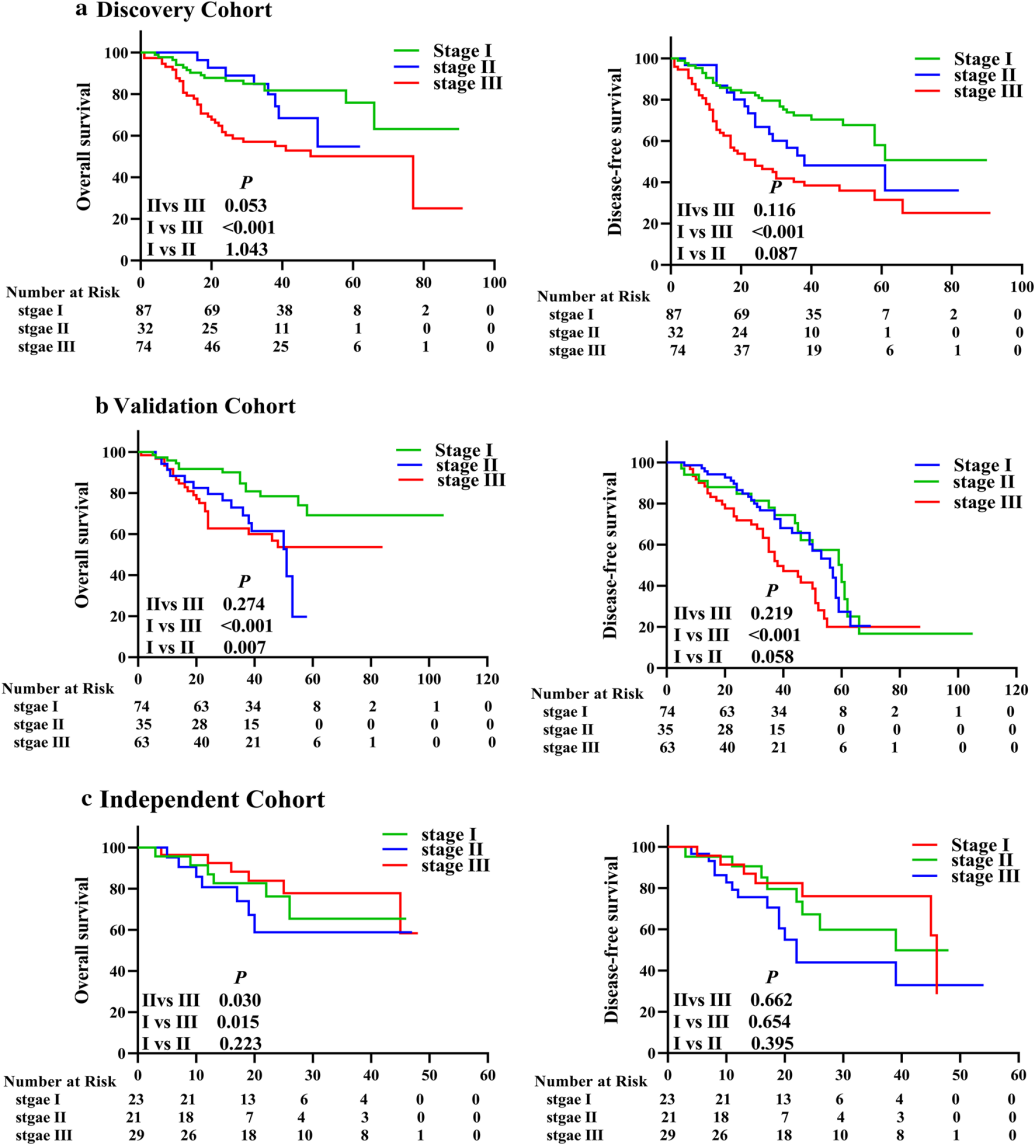
The TNM staging system did not predict survival well in the 3 NSCLC cohorts. The TNM staging system is the main tool for predicting survival and determining the treatment strategies, but it did not predict survival well for patients with NSCLC. The Kaplan–Meier survival curves for OS and DFS of patients with stage I, II, or III NSCLC in **a** the discovery cohort (n = 194), **b** the validation cohort (n = 172), and **c** the independent cohort (n = 73) are shown

**Fig. 5:**
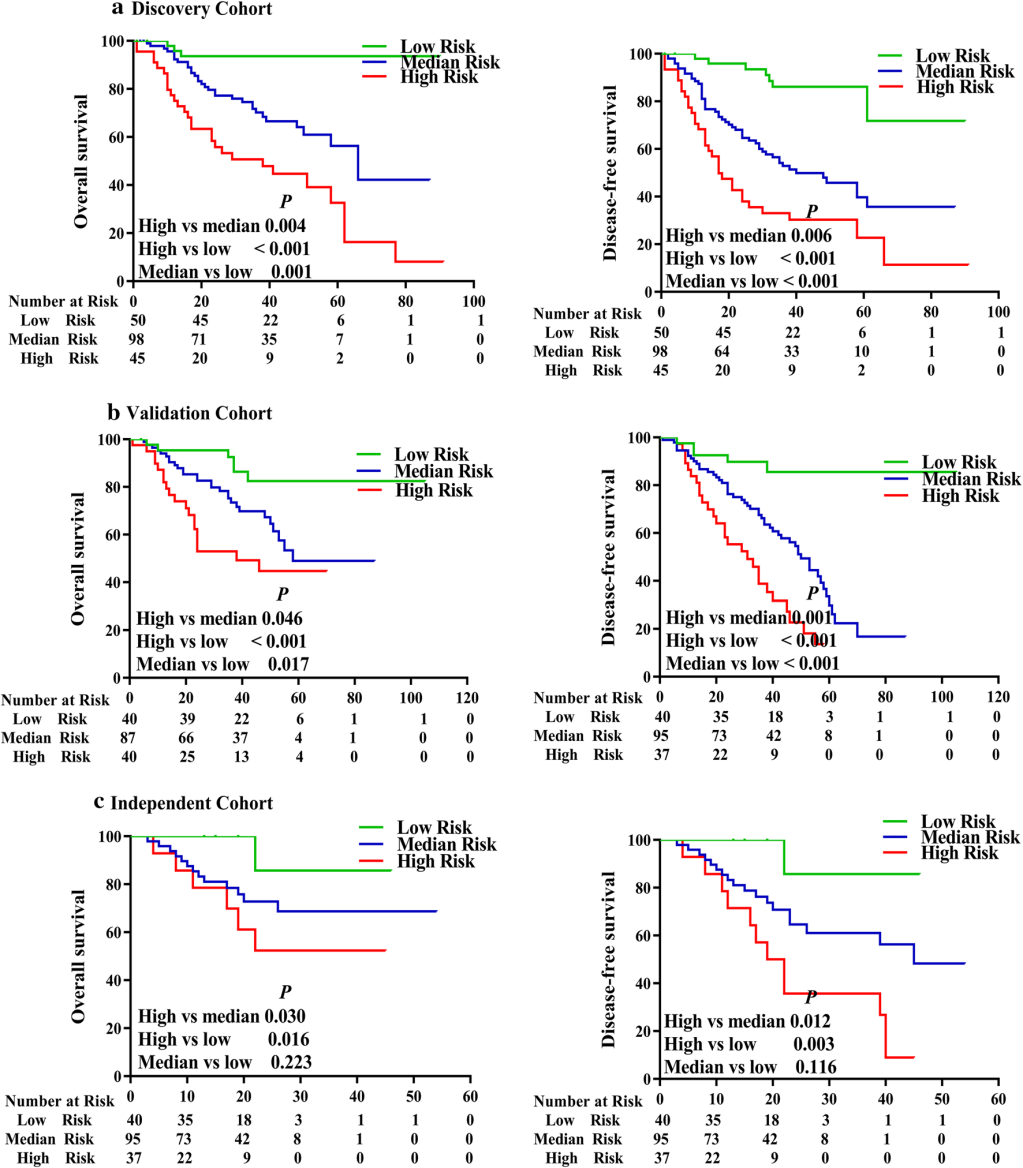
The prognostic value of the combination of the 4-lnRNA signature and TNM stage in the 3 NSCLC cohorts. To improve the TNM staging system, the 4-lnRNA signature is combined with TNM stage to construct a new risk model for predicting survival in patients with NSCLC. According to the new risk score, patients were categorized into low-, medium-, and high-risk groups. Then Kaplan–Meier survival analysis was used to compare the OS and DFS of patients with low, medium, or high risk in **a** the discovery cohort, **b** the internal validation cohort, and **c** the independent validation cohort

In clinical practice, the traditional TNM staging system is the main assessment used to predict the survival of patients with NSCLC and to determine the treatment strategy. However, the TNM staging system is mainly based on anatomical information and does not include factors related to the tumor biology. Therefore, the TNM system is insufficient for predicting survival outcomes in patients with NSCLC [30]. For example, Kaplan–Meier-survival analysis of the 3 cohorts in this study showed that the TNM stage system did not effectively determine the prognosis of NSCLC patients at different stages, especially in stages I and II (Fig. 4). To improve the ability of the TNM staging system to predict patient survival, we established a new risk-score model by combining the risk scores of the 4-lncRNA signature and the TNM staging system: low- and high-risk signatures were scored as 0 and 1, respectively, and stage I, II, and III NSCLC were scored as 1, 2, and 3, respectively. Patients with combined scores of 1, 2–3, or 4 were classified as low-, medium- or high-risk patients, respectively. Then we performed Kaplan–Meier-survival analysis of the patients with different combined risks in the 3 cohorts. The results revealed significant differences in OS and DFS rates between patients with low, medium, or high risk in the discovery cohort (Fig. 5a), and these results were confirmed in the internal validation and independent validation cohorts (Fig. 5b, c).

**Fig. 6:**
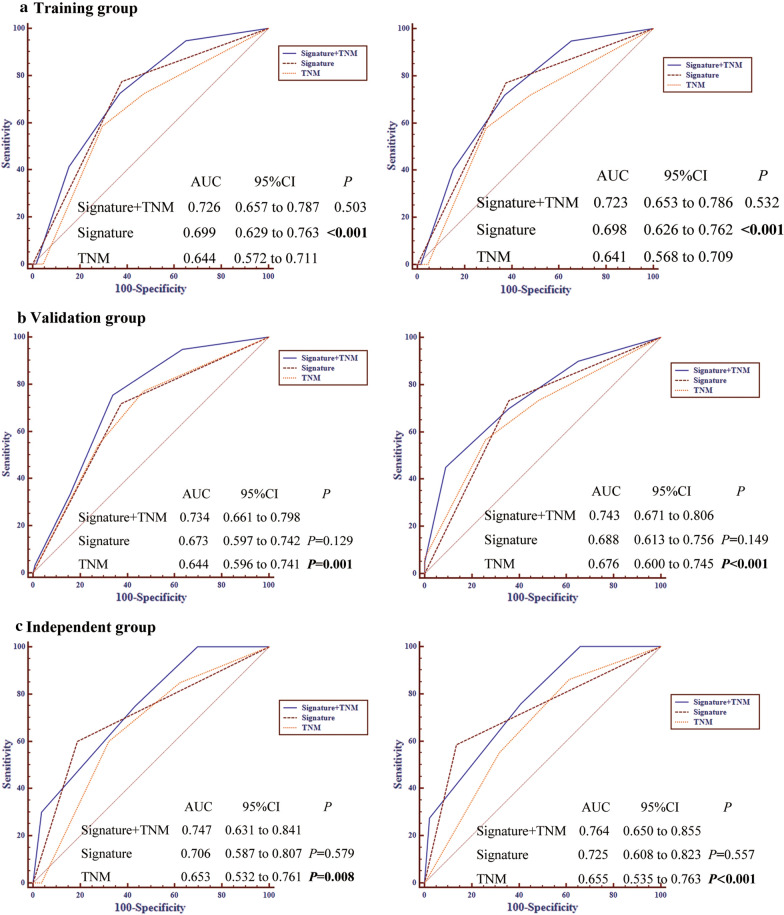
The combined prognostic model is significantly better than the TNM staging system alone in predicting the survival of patients with NSCLC. ROC analysis was employed to compare the predictive accuracy of the three survival predictors including 4-lncRNA signature, the TNM stage and the combined model. A comparison of the three survival predictors in predicting OS (left panel) and DFS (right panel) in the discovery cohort (**a**), internal validation cohort (**b**) and independent validation cohort (**c**) is shown

Next, receiver operating characteristic (ROC) analysis was performed to compare the accuracy of the TNM staging system and the combined-risk model. ROC analysis showed that the combined-risk model achieved a significantly higher predictive accuracy for OS (AUC = 0.726 vs. 0.644) and DFS (AUC = 0.723 vs. 0.641) than that achieved by the TNM staging system in the discovery cohort (Fig. 6a). Similar results were observed in the internal validation cohort and the independent validation cohort (Fig. 6b, c). These results demonstrated that the 4-lncRNA signature can provide additional prognostic information and improve the prognostic power of the TNM staging system.

## Discussion

LncRNAs are widely dysregulated in various cancers and participate in a diverse range of associated biological functions. Numerous aberrant lncRNAs have been detected as hallmarks of cancers and can potentially be used for diagnosis, prognosis, and targeted therapy in cancer. Some investigators have discovered lncRNA profiles and lncRNA signatures in NSCLC by mining data from the GEO and TCGA databases. For example, Zhou et al [31] analyzed the lncRNA-expression profiles of 603 patients from 3 independent NSCLC cohorts in the GEO database and developed a risk-score model based on the expression of 8 lncRNAs, which were significantly associated with OS in patients with NSCLC. Lin et al. [10] identified a 7-lncRNA signature for predicting the OS of patients with NSCLC after combining lncRNA profiles from 4 GEO datasets and validated the signature in 2 independent datasets (TCGA and GSE31210). Recently, He et al. [32] proposed a novel 8-gene signature as a prognostic indicator for patients with early-stage NSCLC after analyzing data from the GEO and TCGA projects. However, the abovementioned prognostic signatures generated by data mining have not been confirmed in patients with NSCLC in a prospective multicenter study. Therefore, the clinical application of prognostic lncRNA biomarkers in NSCLC remains very limited to date. Here, we report the first lncRNA-expression profiling (as determined by microarray analysis) of a large cohort of patients with NSCLC and the identification of an effective prognostic 4-lncRNA signature.

In this study, we identified 305 aberrantly expressed lncRNAs in 104 NSCLC tissues when compared with those in matched normal tissues in the discovery cohort, using a custom lncRNA microarray containing 2412 probes. Notably, we identified a novel 4-lncRNA prognostic signature for patients with NSCLC in the discovery cohort. Kaplan–Meier-survival analysis demonstrated the effective prognostic performance of the 4-lncRNA signature in all the 3 cohorts. Multivariate Cox-regression analysis identified the 4-lncRNA signature as an independent prognostic factor for patients with NSCLC in all the cohorts.

Although TNM staging is widely accepted for disease prognosis and guiding treatment decisions for most solid cancers (including NSCLC), at present, the TNM staging system has critical limitations and insufficiencies in clinical practice, due to intra-tumoral molecular and genetic heterogeneities among patients with lung cancer. The clinical outcomes of lung cancer patients with similar clinical and pathological features are often quite different after receiving similar treatments. Therefore, more personalized molecular markers are urgently needed to assist doctors in clinical practice. In our stratified analysis, the 4-lncRNA signature showed prognostic value for patients at the same stage. Moreover, a risk-score model derived by combining the 4-lnRNA signature and the TNM stage was developed. The combined risk score showed superior performance in predicting OS and DFS rates in all the 3 cohorts, compared with TNM staging system, based on Kaplan–Meier-survival analysis and ROC analysis. Our findings demonstrated that the 4-lncRNA signature can significantly improve the prognostic accuracy of TNM staging and that it can potentially be considered as a marker for risk assessment among patients with NSCLC. Combining the 4-lncRNA signature with the traditional TNM staging parameters might serve as a powerful prognostic approach for patients with NSCLC and can potentially facilitate the selection of patients with more aggressive disease who would benefit from adjuvant therapy.

Among the 4 lncRNAs in the lncRNA signature, only NEAT1 has been linked with cancer. NEAT1 is aberrantly expressed in many malignant human diseases (including lung cancer) and functions as an oncogene. Higher NEAT1 expression correlated with an advanced TNM stage and lymphatic metastasis in patients with NSCLC [33]. Previous findings revealed that NEAT1 promoted the epithelial–mesenchymal transition and metastasis in NSCLC via the Wnt/β-catenin pathway [25, 34]. However, the association of NEAT1 with the survival of patients with lung cancer has not been reported previously. Consistent with published reports, we found that NEAT1 expression was significantly higher in NSCLC tissues than in adjacent normal tissues (fold-change = 1.7). Moreover, we found the first evidence that NEAT1 can serve as an independent prognostic indicator for patients with NSCLC (unpublished data). To our knowledge, the remaining 3 lncRNAs (lnc-GAN1, ASLNC11245, and GSO_1539832_023) in the prognostic 4-lncRNA signature have not been functionally annotated. In our study, these 3 lncRNAs were significantly down-regulated in lung cancer tissues compared with adjacent normal tissues (fold-change = 0.39, 0.75, and 0.47, respectively), and high expression levels of these lncRNAs could serve as indicators for a good prognosis of patients with NSCLC.

Current treatment strategies for lung cancer have led to a comprehensive approach that includes surgery, radiotherapy, chemotherapy, targeted therapy, gene therapy, and immunotherapy [35, 36]. Based on insights gained into the molecular mechanisms underlying NSCLC in the past 10 years, common mutations in genes encoding EGFR-TKIs (EGFR tyrosine kinase inhibitors), programmed cell death protein 1, and members of the epidermal growth factor receptor super-family have been treated clinically with targeted tyrosine-kinase inhibitors [37–43]. Even though these targeted therapies have improved the survival rates and quality of life of patients with NSCLC, their effects are far from satisfactory. Most patients exhibit drug resistance or disease progression after receiving treatment for a certain period of time [44, 45]. Therefore, specific biomarkers for monitoring therapeutic responses in patients with NSCLC are urgently needed. By applying microarray and RNA-seq technology in cancer research, numerous molecular biomarkers have been identified that can predict the responses to specific treatment regimens [46–48]. Of the 4-lncRNA signature identified in this study, NEAT1 was significantly up-regulated in paclitaxel-resistant NSCLC cells and contributed to paclitaxel resistance by activating the Akt/mTOR-signaling pathway [49]. Recent data showed that NEAT1 can inhibit apoptosis in multiple myeloma cells by regulating genes involved in DNA-repair processes, including the homologous-recombination pathway, suggesting its association with drug resistance [49]. Therefore, NEAT1, a component of our 4-lncRNA signature, may play an important role in NSCLC.

Although the 4-lncRNA prognostic signature is a novel and potentially powerful predictor for survival in NSCLC patients, further prospective validation studies in larger cohorts and clinical trials are still required. This study also has other limitations. First, although the 4-lncRNA signature was identified in a large number of NSCLC samples from 2 different regions of China, the signature still needs to be validated in a larger prospective multicenter study, involving patients from more institutions and other countries. Second, the efficacy of models based on multiple types of markers are thought to provide better prognostic value than a single type of marker. Thus, further study will be conducted to identify a multi-gene panel by integrating lncRNAs, microRNAs, and messenger RNAs, with the aim of obtaining a more accurate prognostic assessment of NSCLC. Finally, further experiments need to be performed to elucidate the characteristics and functions of the identified prognostic lncRNAs.

## Conclusions

In this study, our findings reveal a tumor-specific lncRNA expression profile in NSCLC tissues and a novel prognostic signature based on 4 lncRNAs, which is a powerful and independent predictor of OS and DFS in patients with NSCLC. Moreover, a new prognostic model is developed by combining the 4-lncRNA signature and TNM stage to refine the current staging system and to improve the predictive performance. The results of our study suggest that the 4-lncRNA classifier might serve as a precise predictive biomarker for selecting high-risk patients who might benefit from adjuvant therapy and thus guide the personalized management of patients with NSCLC.

## Supplementary information

**Additional file 1.** Additional 2 tables and 1 figures.

## Data Availability

All data in our study are available upon request.

## Abbreviations

ADC: Adenocarcinoma
DFS: Disease-free survival
FDR: False-discovery rate
GEO: Gene Expression Omnibus
IQR: Inter-quartile range
lncRNA: Long non-coding RNA
NSCLC: Non-small cell lung carcinoma
OS: Overall survival
qRT-PCR: Quantitative reverse transcriptase-polymerase chain reaction
RNA-seq: RNA-sequencing
ROC: Receiver operating characteristic
SAM: Significance Analysis of Microarrays
SCC: Squamous cell carcinoma
SD: Standard deviation
SDS: Sodium dodecyl sulfate
SSC: Saline-sodium citrate
TCGA: The Cancer Genome Atlas
TNM: Tumor, nodes, and metastases
