# Supplementary material for: Identification of a 4-lncRNA signature predicting prognosis of patients with non-small cell lung cancer: a multicenter study in China
Source: J Transl Med. 2020 Aug 20;18:320. doi: 10.1186/s12967-020-02485-8 (PMC7441565; doi:10.1186/s12967-020-02485-8)
Supplement: Supplementary file 1 — Additional file 1. Additional 2 tables and 1 figures. [file 12967_2020_2485_MOESM1_ESM.docx]

**Additional file**

**I****dentification of a 4-lncRNA** **Signature Predicting Prognosis of Patients with Non-Small Cell Lung Cancer: a Multicenter Study in China**

Rui-Qi Wang^1#^, Xiao-Ran Long^1,2#^, Chun-Lei Ge^3#^, Mei-Yin Zhang^1^, Long Huang^4^, Ning-Ning Zhou^5^, Yi Hu^1,6^, Rui-Lei Li^3^, Zhen Li^3^, Dong-Ni Chen^1,6^, Lan-Jun Zhang^1,6^, Zhe-Sheng Weng^1,6^, Shi-Juan Mai^1*^, Hui-Yun Wang^1*^

# 1. Additional Tables: 2 tables

**2. Additional** **Figures: 1 figures**

**Table S1. The** **primer sequences of five lncRNAs for qRT-PCR.**

| **Primer name** | **sequence** |
| --- | --- |
| NEAT1-F | 5-CCTGCCTTCTTGTGCGTTTC-3 |
| NEAT1-R | 5-CTTGTACCCTCCCAGCGTTT-3 |
| GAN1-F | 5-GACAGTGTTGGCAAGAACGG-3 |
| GAN1-R | 5-CTTGCCCAGCACTCTTCTTTG-3 |
| XLOC_001306-F | 5-GTCCGTGAAAAGTGATGGCA-3 |
| XLOC_001306-R | 5-GTGACTGGGAACAGTCGCAA-3 |
| XLOC_005302-F | 5-CCTTGCTTCTGTGGTCTGGT-3 |
| XLOC_005302-R | 5-TGGCCCCTACAAACTCCAAC-3 |
| XLOC_009261-F | 5-AAAGGAAAAGTGGCACGGC-3 |
| XLOC_009261-R | 5-TCAAGCCCAGACTCTCAGTG-3 |

-F: forward primer; -R: reverse primer.

| **Table S2 .List of the 305 Significantly Deferentially Expressed lncRNAs** | | | | | | | |  |
| --- | --- | --- | --- | --- | --- | --- | --- | --- |
| **No.** | **Gene Name** | **Fold Change** | **q-value (%)** | **No.** | **Gene Name** | **Fold Change** | **q-value (%)** | |
|  | **Upregulated genes** |  |  |  |  |  |  | |
| **1** | XLOC_001447 | 1.83 | 0 | **49** | ASLNC17263 | 1.45 | 0 | |
| **2** | CK327199 | 2.20 | 0 | **50** | BE072494 | 1.31 | 0 | |
| **3** | XLOC_002977 | 1.95 | 0 | **51** | BF894809 | 1.33 | 0 | |
| **4** | XLOC_003292 | 1.53 | 0 | **52** | XLOC_006886 | 1.29 | 0 | |
| **5** | XLOC_009302 | 2.36 | 0 | **53** | snaR | 1.34 | 0 | |
| **6** | BF894926 | 1.46 | 0 | **54** | BE156015 | 1.25 | 0 | |
| **7** | BF942971 | 2.23 | 0 | **55** | BQ321884 | 1.43 | 0 | |
| **8** | BE008080 | 1.47 | 0 | **56** | TEA ncRNAs | 1.31 | 0 | |
| **9** | **NEAT1** | **1.70** | 0 | **57** | ASLNC03406 | 1.33 | 0 | |
| **10** | XLOC_000318 | 1.43 | 0 | **58** | BF754532 | 1.37 | 0 | |
| **11** | XLOC_005341 | 1.41 | 0 | **59** | BE700386 | 1.44 | 0 | |
| **12** | ASLNC03356 | 2.53 | 0 | **60** | BE181839 | 1.26 | 0 | |
| **13** | CK327060 | 2.32 | 0 | **61** | ASLNC21327 | 1.28 | 0 | |
| **14** | XLOC_014001 | 1.35 | 0 | **62** | BF986931 | 1.36 | 0 | |
| **15** | XLOC_001435 | 1.53 | 0 | **63** | BE062769 | 1.40 | 0 | |
| **16** | XLOC_005046 | 1.49 | 0 | **64** | AW902271 | 1.44 | 0 | |
| **17** | GRM4 | 1.82 | 0 | **65** | BF805741 | 1.48 | 0 | |
| **18** | XLOC_007131 | 1.35 | 0 | **66** | ASLNC00530 | 1.30 | 0 | |
| **19** | XLOC_014287 | 1.28 | 0 | **67** | AW995014 | 1.31 | 0 | |
| **20** | LOC285194 | 1.69 | 0 | **68** | CK327114 | 1.25 | 0 | |
| **21** | XLOC_001771 | 1.48 | 0 | **69** | ASLNC06829 | 1.28 | 0 | |
| **22** | BF364433 | 1.43 | 0 | **70** | GSO_1539211_161 | 1.35 | 0 | |
| **23** | GSO_1539211_523 | 1.30 | 0 | **71** | ASLNC06809 | 1.26 | 0 | |
| **24** | AW851934 | 1.35 | 0 | **72** | BF805156 | 1.25 | 0 | |
| **25** | ASLNC22620 | 1.45 | 0 | **73** | NEIF3D | 1.32 | 0 | |
| **26** | ASLNC14708 | 1.27 | 0 | **74** | BE069131 | 1.33 | 0 | |
| **27** | XLOC_006933 | 1.45 | 0 | **75** | GSO_1539211_373 | 1.25 | 0 | |
| **28** | GSO_1539211_446 | 1.44 | 0 | **76** | GSO_1539211_468 | 1.41 | 0 | |
| **29** | GSO_1539211_543 | 1.43 | 0 | **77** | XLOC_011110 | 1.33 | 0 | |
| **30** | BF898705 | 1.28 | 0 | **78** | GSO_1539211_351 | 1.99 | 0 | |
| **31** | XLOC_000529 | 1.34 | 0 | **79** | GSO_1539211_121 | 1.25 | 0 | |
| **32** | EXO-2 | 1.37 | 0 | **80** | ASLNC04893 | 1.34 | 0 | |
| **33** | HOXA6as | 1.42 | 0 | **81** | GSO_1539211_368 | 1.34 | 0 | |
| **34** | XLOC_011213 | 1.47 | 0 | **82** | BF881796 | 1.32 | 0 | |
| **35** | ASLNC08303 | 1.82 | 0 | **83** | XLOC_005849 | 1.44 | 0 | |
| **36** | XLOC_001208 | 1.31 | 0 | **84** | ASLNC11164 | 1.30 | 0 | |
| **37** | BQ376030 | 1.35 | 0 | **85** | Recombination hot spot RNA | 1.29 | 0 | |
| **38** | EGO | 1.49 | 0 | **86** | GSO_1539211_395 | 1.27 | 0 | |
| **39** | PANDA | 1.39 | 0 | **87** | XLOC_003872 | 1.25 | 0 | |
| **40** | XLOC_007264 | 1.55 | 0 | **88** | BE003587 | 1.33 | 0 | |
| **41** | XLOC_011486 | 1.41 | 0 | **89** | XLOC_005902 | 1.25 | 0 | |
| **42** | AW797125 | 1.35 | 0 | **90** | AW995549 | 1.31 | 0 | |
| **43** | BF363237 | 1.44 | 0 | **91** | B2 SINE RNA | 1.31 | 0 | |
| **44** | GSO_1539296_067 | 1.28 | 0 | **92** | BF368525 | 1.29 | 0 | |
| **45** | CDR1 AS | 1.27 | 0 | **93** | GSO_1539211_352 | 1.39 | 0 | |
| **46** | XLOC_013770 | 1.54 | 0 | **94** | AW845241 | 1.37 | 0 | |
| **47** | BE696363 | 1.36 | 0 | **95** | XLOC_010207 | 1.25 | 0 | |
| **48** | XLOC_011978 | 1.31 | 0 | **96** | AW937492 | 1.28 | 0 | |

**Table S2 continue 1：**

| **97** | XLOC_012799 | 1.25 | 0 | **11** | **GSO_1539832_023** | **0.47** | **0** |
| --- | --- | --- | --- | --- | --- | --- | --- |
| **98** | ASLNC06184 | 1.28 | 0 | **12** | GSO_1539211_350 | 0.37 | 0 |
| **99** | BF918740 | 1.33 | 0 | **13** | GSO_1539211_452 | 0.43 | 0 |
| **100** | CK327086 | 1.26 | 0 | **14** | GSO_1539211_316 | 0.71 | 0 |
| **101** | ASLNC14646 | 1.28 | 0 | **15** | GSO_1539211_465 | 0.42 | 0 |
| **102** | ASLNC14489 | 1.33 | 0 | **16** | BF848216 | 0.66 | 0 |
| **103** | XLOC_002897 | 1.25 | 0 | **17** | CK327073 | 0.71 | 0 |
| **104** | GSO_1539211_335 | 1.33 | 0 | **18** | GSO_1539296_124 | 0.63 | 0 |
| **105** | BE007897 | 1.25 | 0 | **19** | GSO_1539211_039 | 0.73 | 0 |
| **106** | AW805354 | 1.25 | 0 | **20** | BE007836 | 0.71 | 0 |
| **107** | BE080898 | 1.33 | 0 | **21** | ASLNC21391 | 0.68 | 0 |
| **108** | GSO_1539211_136 | 1.29 | 0 | **22** | CK327133 | 0.64 | 0 |
| **109** | GSO_1539211_369 | 1.31 | 0 | **23** | GSO_1539211_002 | 0.74 | 0 |
| **110** | GSO_1539211_519 | 1.36 | 0 | **24** | GSO_1539211_040 | 0.75 | 0 |
| **111** | BC200 | 1.31 | 0 | **25** | GSO_1539296_048 | 0.69 | 0 |
| **112** | Rian | 1.28 | 0 | **26** | BE071515 | 0.62 | 0 |
| **113** | CK327129 | 1.25 | 0 | **27** | GSO_1539296_148 | 0.69 | 0 |
| **114** | UBC | 1.31 | 0 | **28** | GSO_1539211_027 | 0.78 | 0 |
| **115** | GSO_1539211_357 | 1.38 | 0 | **29** | GSO_1539296_080 | 0.76 | 0 |
| **116** | GSO_1539211_025 | 1.26 | 0 | **30** | GSO_1539296_090 | 0.73 | 0 |
| **117** | ASLNC04230 | 1.33 | 0 | **31** | ASLNC13543 | 0.77 | 0 |
| **118** | XLOC_011976 | 1.28 | 0 | **32** | ASLNC14767 | 0.77 | 0 |
| **119** | XLOC_005087 | 1.27 | 0 | **33** | BE146492 | 0.68 | 0 |
| **120** | BF350736 | 1.28 | 0 | **34** | GSO_1539296_105 | 0.70 | 0 |
| **121** | BG009898 | 1.28 | 0 | **35** | GSO_1539211_011 | 0.80 | 0 |
| **122** | CK327112 | 1.25 | 0 | **36** | GSO_1539211_436 | 0.74 | 0 |
| **123** | CK327190 | 1.26 | 0 | **37** | AW937741 | 0.71 | 0 |
| **124** | BF364175 | 1.28 | 0 | **38** | ASLNC03674 | 0.48 | 0 |
| **125** | CK327081 | 1.32 | 0 | **39** | ASLNC02611 | 0.77 | 0 |
| **126** | XLOC_013670 | 1.29 | 0 | **40** | XLOC_010897 | 0.68 | 0 |
| **127** | GSO_1539211_314 | 1.50 | 0 | **41** | GSO_1539211_422 | 0.78 | 0 |
| **128** | 21A | 1.31 | 0 | **42** | GSO_1539296_139 | 0.75 | 0 |
| **129** | XLOC_004243 | 1.31 | 0 | **43** | GSO_1539211_378 | 0.67 | 0 |
| **130** | ASLNC21300 | 1.33 | 0 | **44** | GSO_1539211_374 | 0.59 | 0 |
| **131** | CK327033 | 1.33 | 0 | **45** | GSO_1539296_173 | 0.75 | 0 |
| **132** | BF363796 | 1.26 | 0 | **46** | BF999168 | 0.77 | 0 |
| **133** | GSO_1539211_281 | 1.32 | 0 | **47** | ASLNC01041 | 0.76 | 0 |
| **134** | BE069210 | 1.34 | 0 | **48** | GSO_1539211_005 | 0.76 | 0 |
| **135** | XLOC_009437 | 1.26 | 0 | **49** | HOTAIRM1 | 0.75 | 0 |
| **136** | XLOC_000194 | 1.30 | 0 | **50** | GSO_1539211_026 | 0.74 | 0 |
| **137** | ncR-uPAR | 1.41 | 0 | **51** | ASLNC14270 | 0.72 | 0 |
| **138** | GSO_1539211_596 | 1.55 | 0 | **52** | GSO_1539296_163 | 0.72 | 0 |
|  | **Downregulated Gene** |  |  | **53** | GSO_1539211_220 | 0.76 | 0 |
| **1** | GSO_1539211_376 | 0.37 | 0 | **54** | ASLNC05193 | 0.64 | 0 |
| **2** | ASLNC04697 | 0.50 | 0 | **55** | BF363576 | 0.72 | 0 |
| **3** | GSO_1539211_070 | 0.52 | 0 | **56** | Emx2os | 0.71 | 0 |
| **4** | **Lnc-GAN1** | **0.39** | 0 | **57** | ASLNC10399 | 0.73 | 0 |
| **5** | GSO_1539211_206 | 0.68 | 0 | **58** | GSO_1539296_129 | 0.73 | 0 |
| **6** | ASLNC11225 | 0.51 | 0 | **59** | BE061008 | 0.71 | 0 |
| **7** | ASLNC24740 | 0.57 | 0 | **60** | BF328128 | 0.72 | 0 |
| **8** | BF892893 | 0.66 | 0 | **61** | AW753003 | 0.76 | 0 |
| **9** | ASLNC09418 | 0.68 | 0 | **62** | GSO_1539211_440 | 0.72 | 0 |
| **10** | GSO_1539296_177 | 0.65 | 0 | **63** | BF908905 | 0.64 | 0 |

**Table S2 continue 2:**

| **64** | **ASLNC11245** | **0.75** | 0 | **116** | GSO_1539211_036 | 0.79 | 0 |
| --- | --- | --- | --- | --- | --- | --- | --- |
| **65** | GSO_1539211_392 | 0.60 | 0 | **117** | ASLNC03327 | 0.70 | 0 |
| **66** | GSO_1539211_095 | 0.75 | 0 | **118** | GSO_1539211_472 | 0.67 | 0 |
| **67** | ASLNC14532 | 0.77 | 0 | **119** | GSO_1539211_338 | 0.79 | 0 |
| **68** | BF363687 | 0.74 | 0 | **120** | ASLNC09821 | 0.79 | 0 |
| **69** | GSO_1539296_195 | 0.76 | 0 | **121** | ASLNC11284 | 0.79 | 0 |
| **70** | XLOC_013095 | 0.80 | 0 | **122** | GSO_1539211_029 | 0.75 | 0 |
| **71** | GSO_1539211_048 | 0.79 | 0 | **123** | ASLNC16202 | 0.70 | 0 |
| **72** | ASLNC04536 | 0.71 | 0 | **124** | ASLNC12371 | 0.72 | 0 |
| **73** | ASLNC15995 | 0.74 | 0 | **125** | GSO_1539211_303 | 0.73 | 0 |
| **74** | ASLNC10102 | 0.75 | 0 | **126** | GSO_1539211_401 | 0.80 | 0 |
| **75** | GSO_1539296_120 | 0.69 | 0 | **127** | ASLNC12376 | 0.75 | 0 |
| **76** | AW996899 | 0.74 | 0 | **128** | ASLNC06741 | 0.74 | 0 |
| **77** | ASLNC24052 | 0.72 | 0 | **129** | ASLNC09272 | 0.74 | 0 |
| **78** | XLOC_006980 | 0.77 | 0 | **130** | GSO_1539296_088 | 0.75 | 0 |
| **79** | ASLNC20113 | 0.76 | 0 | **131** | GSO_1539211_104 | 0.79 | 0 |
| **80** | XLOC_000942 | 0.79 | 0 | **132** | ASLNC19507 | 0.73 | 0 |
| **81** | GSO_1539296_151 | 0.72 | 0 | **133** | GSO_1539296_087 | 0.74 | 0 |
| **82** | GSO_1539211_102 | 0.72 | 0 | **134** | ASLNC02009 | 0.75 | 0 |
| **83** | ASLNC00555 | 0.70 | 0 | **135** | GSO_1539832_027 | 0.74 | 0 |
| **84** | ASLNC21884 | 0.74 | 0 | **136** | ASLNC11181 | 0.76 | 0 |
| **85** | BF849127 | 0.70 | 0 | **137** | GSO_1539296_126 | 0.72 | 0 |
| **86** | ASLNC20011 | 0.75 | 0 | **138** | GSO_1539211_054 | 0.72 | 0 |
| **87** | GSO_1539211_534 | 0.75 | 0 | **139** | ASLNC18559 | 0.74 | 0 |
| **88** | GSO_1539211_035 | 0.73 | 0 | **140** | GSO_1539211_425 | 0.70 | 0 |
| **89** | GSO_1539296_081 | 0.72 | 0 | **141** | ASLNC20064 | 0.79 | 0 |
| **90** | ASLNC06639 | 0.72 | 0 | **142** | GAS5 | 0.78 | 0 |
| **91** | GSO_1539296_100 | 0.72 | 0 | **143** | GSO_1539296_135 | 0.75 | 0 |
| **92** | BE156454 | 0.73 | 0 | **144** | GSO_1539211_086 | 0.72 | 0 |
| **93** | BF357721 | 0.76 | 0 | **145** | ASLNC03183 | 0.76 | 0 |
| **94** | XLOC_000236 | 0.72 | 0 | **146** | XLOC_000095 | 0.79 | 0 |
| **95** | XLOC_001704 | 0.74 | 0 | **147** | GSO_1539296_085 | 0.71 | 0 |
| **96** | GSO_1539211_051 | 0.69 | 0 | **148** | BF364140 | 0.80 | 0 |
| **97** | ASLNC18594 | 0.73 | 0 | **149** | AW995669 | 0.75 | 0 |
| **98** | ASLNC10710 | 0.70 | 0 | **150** | XLOC_001641 | 0.77 | 0 |
| **99** | XLOC_002530 | 0.73 | 0 | **151** | AW846670 | 0.71 | 0 |
| **100** | BF368528 | 0.71 | 0 | **152** | ASLNC17389 | 0.75 | 0 |
| **101** | GSO_1539211_439 | 0.60 | 0 | **153** | ASLNC06990 | 0.78 | 0 |
| **102** | XLOC_010990 | 0.73 | 0 | **154** | GSO_1539296_010 | 0.72 | 0 |
| **103** | GSO_1539296_146 | 0.70 | 0 | **155** | GSO_1539832_036 | 0.73 | 0 |
| **104** | ASLNC18688 | 0.77 | 0 | **156** | GSO_1539296_029 | 0.68 | 0 |
| **105** | GSO_1539832_035 | 0.70 | 0 | **157** | ASLNC18858 | 0.79 | 0 |
| **106** | ASLNC23197 | 0.60 | 0 | **158** | ASLNC12773 | 0.74 | 0 |
| **107** | ASLNC13096 | 0.73 | 0 | **159** | AW880165 | 0.74 | 0 |
| **108** | ASLNC04817 | 0.72 | 0 | **160** | ASLNC09137 | 0.75 | 0 |
| **109** | GSO_1539211_001 | 0.73 | 0 | **161** | ASLNC11403 | 0.73 | 0 |
| **110** | GSO_1539211_245 | 0.77 | 0 | **162** | Zfhx2as | 0.72 | 0 |
| **111** | ASLNC03644 | 0.72 | 0 | **163** | GSO_1539211_053 | 0.72 | 0 |
| **112** | GSO_1539211_175 | 0.75 | 0 | **164** | GSO_1539211_394 | 0.77 | 0 |
| **113** | BF368747 | 0.73 | 0 | **165** | ASLNC01820 | 0.72 | 0 |
| **114** | XLOC_009925 | 0.73 | 0 | **166** | GSO_1539211_602 | 0.72 | 0 |
| **115** | ASLNC04585 | 0.71 | 0 | **167** | GSO_1539296_137 | 0.77 | 0 |

**
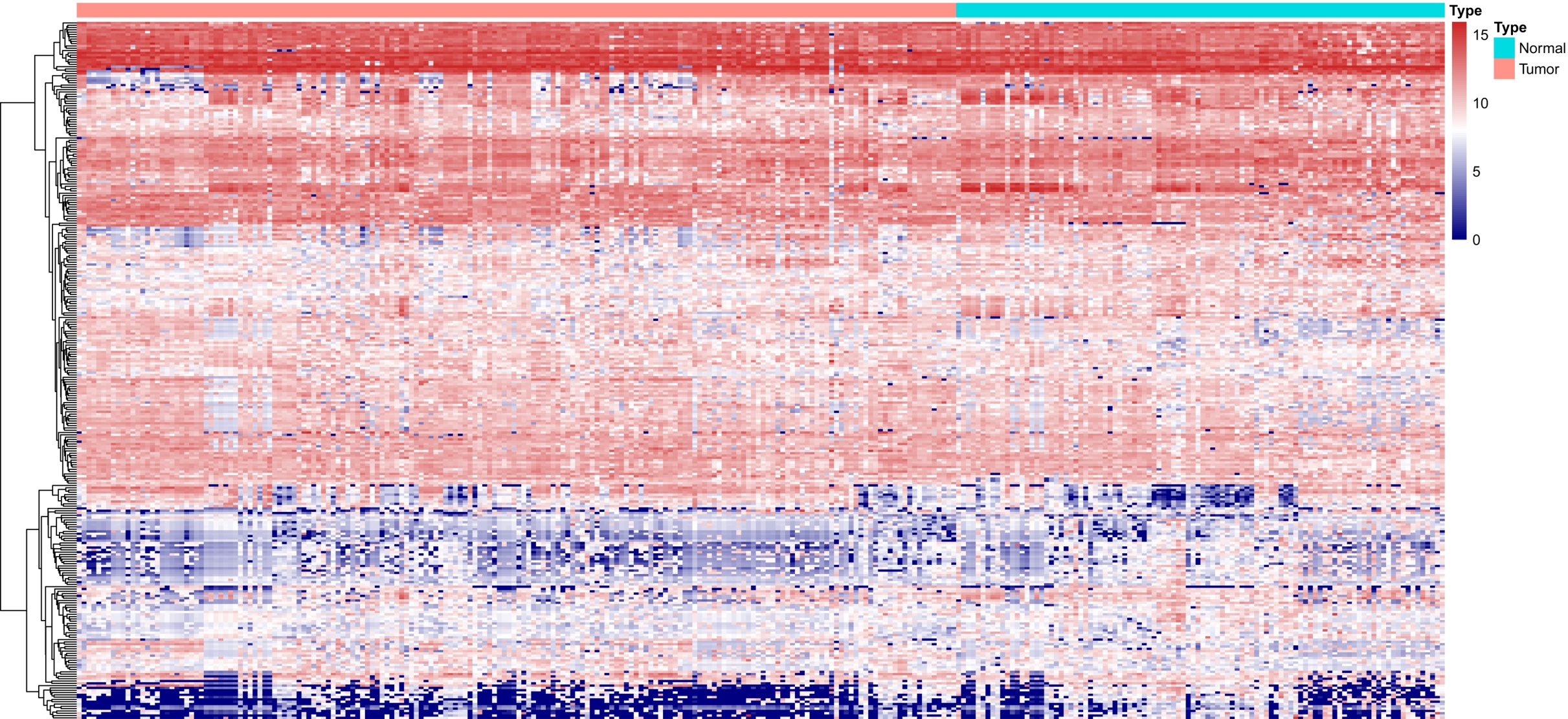
**

**Figure S1. Heatmap of 305 differentially expressed lncRNAs between 194 NSCLC tissues and 100 normal lung samples.**
